# Injury Source and Correlation Analysis of Riders in Car-Electric Bicycle Accidents

**DOI:** 10.1155/2018/3674858

**Published:** 2018-04-08

**Authors:** Tiefang Zou, Liang Yi, Ming Cai, Lin Hu, Yuelin Li

**Affiliations:** ^1^School of Automobile and Mechanical Engineering, Changsha University of Science and Technology, Changsha 410076, China; ^2^Key Laboratory of Safety Design and Reliability Technology for Engineering Vehicle, Hunan Province 410004, China; ^3^School of Engineering, Sun Yat-sen University, Guangzhou, Guangdong 510275, China

## Abstract

The knowledge about the injury source and correlation of riders in car-electric bicycle accident will be helpful in the cross validation of traces and vehicle safety design. In order to know more information about such kind of knowledge, 57 true car-electric bicycle accidents were reconstructed by PC-Crash and then data on injury information of riders were collected directly from the reconstructed cases. These collected data were validated by some existing research results firstly, and then 4 abnormal cases were deleted according to the statistical method. Finally, conclusions can be obtained according to the data obtained from the remaining 53 cases. Direct injuries of the head and right leg are from the road pavement upon low speed; the source laws of indirect head injuries are not obvious. Upon intermediate and high speed, the injuries of the above parts are from automobiles. Injuries of the left leg, femur, and right knee are from automobiles; left knee injuries are from automobiles, the road pavement and automobiles, respectively, upon low, intermediate, and high speed. The source laws of indirect torso injuries are not obvious upon intermediate and low speed, which are from automobiles upon high speed, while direct torso injuries are from the road pavement. And there is no high correlation between all parts of the injury of riders. The largest correlation coefficient was the head-left femur and left femur-right femur, which was 0.647, followed by the head-right femur (0.638) and head-torso which was 0.617.

## 1. Introduction

According to statistics, the total number of traffic accidents in China is declining year by year, but the accidents of car-electric bicycle rise [1]. The number of riders who died in the accident also increased steadily, from 0% in 2000 to 11% [2] in 2014. This shows that it is very necessary for us to study the traffic accidents of car-electric bicycles. Accident reconstruction is an important way to study traffic accidents. According to all kinds of traces that can be obtained in the accident, we can deduce the whole process of an accident, and we can get more data that cannot be measured from the accident scene, such as vehicle speed and human injury [3, 4]. These data are valuable for vehicle safety design, road speed limit setting, and traffic accident identification [5–7]. The basis of accident reconstruction is all kinds of traces left on the scene of the accident. In order to improve the reliability of the reconstruction results, it is often necessary to conduct cross verification [8] to ensure the reliability of the trace. It is noted that there is a certain correspondence between the deformation of the vehicle and the injury of the human body [9, 10]. Therefore, these two kinds of traces can be contrasted and verified. But there is a very important question before the verification; that is, the rider's injury comes from the car or the road. If the problem is unclear, the conclusions are not reliable if verified with the pavement damages and vehicle deformation; at the same time, to find out the source of injury to the rider in the accident, it is also valuable to the safety design of the body, especially the design of the shape of the vehicle head. In addition, the study on the correlation between the injuries of the riders can also provide support for the mutual validation of injury traces. Therefore, the research on the source of human injury has aroused the attention of scholars both in China and abroad: Badea-Rmero and Lenard [11] found that the head injury of a part of riders is caused by the ground based on the accident statistics. With the aid of deep accident investigation, it is found that car collision is the main cause of pedestrian injury, especially serious injury, but when the body is thrown out, the impact with the ground cannot be ignored, and in some cases it can also cause fatal injuries [12]. Chengjian et al. [13] found that the source of injury to the head of pedestrians is related to the speed of the car by way of simulation. When the speed is lower than 30 km/h, the injuries mainly come from the ground; when the speed is larger than 40 km/h, the injuries mainly come from the vehicle. Hua et al. [14] studied the source of head injury in different types of vehicle collision accidents and found that vehicle models have an effect on the source of human injury with the simulation technology. A survey of these studies shows that scholars in China and abroad had studied the source of human injury in pedestrian accidents, but there are few studies on the source of riders' injuries in car-electric bicycle accidents.

In order to know more information about the injury source and correlation of riders in electric bicycle accidents, firstly, 57 car-electric bicycle accidents will be reconstructed by PC-Crash, and then data will be collected and verified by these existing research results; finally, the injury source and correlation of the riders will be studied with the aid of the trend line.

## 2. Source of the Data

57 car-electric bicycle accidents were selected from the Accident Investigation database of Hunan University and the data collected by the authors during the past ten years. Firstly, each accident was reconstructed with PC-Crash; secondly, data on injuries, brake distance, and motion distance of the rider will be collected; thirdly, the existing research results were employed to verify the reliability of these collected data. At the same time, some abnormal values were eliminated with the statistical method, and data of 53 cases were finally obtained.

### 2.1. Accident Reconstruction

In order to obtain the injuries and motion distance of the riders, 57 car-electric bicycle accidents were all reconstructed with PC-Crash. In order to make the reconstruction more reliable, all traces in the accident should be reasonably explained in the process of simulation. One case will be shown as follows to show the process of reconstruction.

#### 2.1.1. Case Introduction

**Figure 1:**
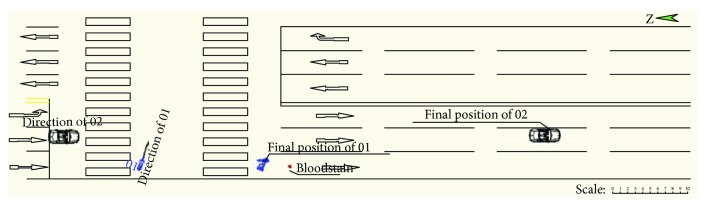
The sketch of the accident scene.

One evening in a city in China, a Chevrolet car ran from north to south and collided with a car-electric bicycle which was running from west to east at the traffic light intersection. According to the survey of the police station, the accident happened on a flat and dry bituminous pavement in good weather and visibility. The vehicle is free of faults. The on-the-spot traces are the rider's blood stain and final stop position of the electric bicycle and car. The braking traces are not discovered.  Figure 1 shows the sketch of the accident scene; vehicle 02 is the Chevrolet car, while vehicle 01 is the electric bicycle. There is no obvious scar on the head of the rider. The rider is conscious but with open fractures on the left leg, three fractures on the rib, and trauma on the right leg.

#### 2.1.2. Accident Reconstruction

According to the existing research [3], a traffic accident can be reconstructed reliably following these steps.

**Figure 2:**
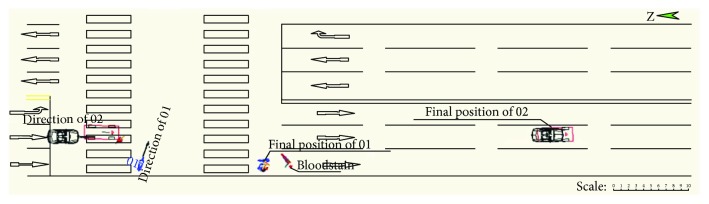
The simulation result.

**Figure 3:**
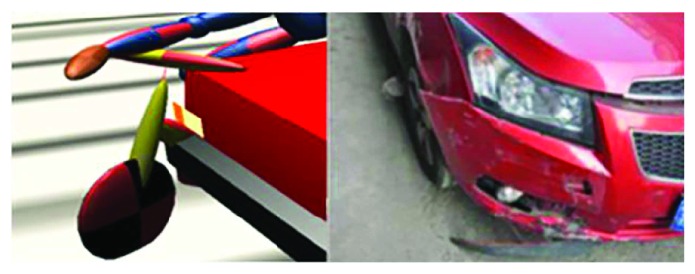
Comparison of the relative position in simulation and the damage of the vehicle at 60 ms.

**Figure 4:**
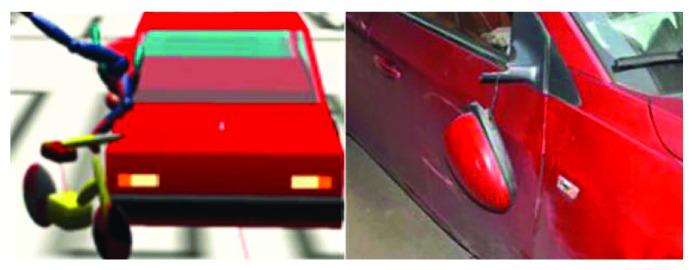
Comparison of the relative position in simulation and the damage of the vehicle at 130 ms.

1. Reconstruct the accident scene. As for this case, the accident happened on a flat asphalt pavement, so there was no need to establish a three-dimensional road. It is only necessary to scale the sketch of the accident scene according to Figure 1 and then place it into PC-Crash.
2. Reconstruct all accident participants. As for the vehicle, directly recall the vehicle model near the accident vehicle from the PC-Crash vehicle database and then modify the parameters with high influence on the reconstruction according to the actual vehicle parameters. After the modification, the car model is characterized by mass 1270 kg, length 4598 mm, width 1797 mm, and height 1470 mm. As for the electric bicycle, the multibody model Maxi-Driver 010910 in PC-Crash was selected and then the corresponding parameters were changed according to the information on the electric bicycle and rider. In the case, the height of the rider is 171 cm and the weight is 70 kg, while for the electric bicycle, the length is 1557 mm, the height of the handle is 960 mm, and the mass is 45 kg. All other parameters are default values.
3. Accident reconstruction. Through repeated simulation, we discovered that it is consistent with the actual condition when the car speed is 64 km/h and the speed of the car-electric bicycle is 3 km/h. The traces in simulation fit with the accident site when the friction coefficient between the car and the pavement is 0.6 and the friction coefficient between the bicycle and the pavement is 0.7. The reconstruction results are shown in Figure 2.
4. Verify the reconstruction result. As shown in Figure 2, traces in the accident scene can be reasonably explained in the simulation. Figures 3 and 4 are the comparison of the relative position in simulation and the damage of the vehicle at the simulation time *t* = 60 ms and *t* = 130 ms. In Figures 3 and 4, the left figure is the reconstruction figure, while the right figure is the actual deformation of the car in the accident. At the reconstruction time *t* = 60 ms, the bicycle contacts with the right front of the car; when *t* = 130 ms, the rider scrubs with the right of the car. These contact points are the causes for the vehicle's deformations. At the same time, Table 1 compares the conjectured conclusions of injuries to the rider and the information provided by the police. It shows that the rider does not suffer serious injuries in addition to the fractures of the left leg. The conclusion is consistent with the information provided by the police. It suggests that the body injuries can be explained reasonably in the simulation also. Thus, the reconstruction results are reliable and the data on this basis are trustable.

### 2.2. Data Reading

All the 57 cases were reconstructed according to the above method. And then the acceleration of the rider's head and chest and the contact force of the head, the torso, the femur, the tibia, and the knee were all derived directly from PC-Crash. And then the time that the rider is high up absolutely in the air will be found from the simulation as the time node. According to the time node, the injury values of different parts of the rider were calculated according to these obtained data. The value at the front of the node is considered from the impact of the vehicle, while the value behind the node is considered from the impact of the road. Detailed information about these data can be found in Tables 2–5.

## 3. Data Processing

### 3.1. Data Verification

**Figure 5:**
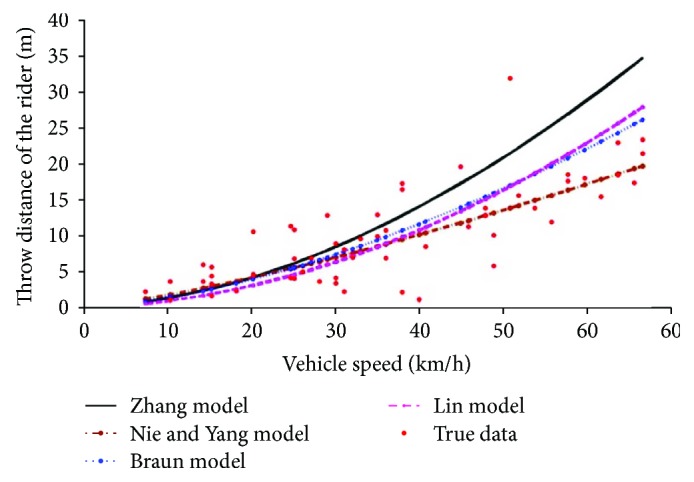
Data verification.

Take the vehicle speed as the *y*-coordinate and the body throw distance as the *x*-coordinate to depict the data, and compare it with existing results. The verification results are shown in Figure 5. More information about Zhang's model [18], Nie and Yang's model [19], Braun's model [20], and Lin et al.'s model [21] can be found in the corresponding references. From Figure 5, we can find that the collected data are evenly distributed around the models proposed by 4 scholars, indicating that the data collected here are reliable and can be further analyzed.

### 3.2. Abnormal Data Processing

In order to reduce the effect of abnormal data on the analysis results, the outliers in these collected data should be deleted before analyzing the data. And the Pauta principle (3*σ*) in statistics was employed to get rid of outliers in the paper.

HIC15 was taken as an example here. Firstly, roughly screen out the value that deviates from most of the values in all cases with a boxplot module in the SPSS software; the corresponding observed values are 5939, 1935, 3337, 1849, and 2300. And then the analyzed results are shown in Table 6 according to the Pauta principle. From Table 6, we can find that the observed value 5939 is the outlier; in this case, it shall be removed. Respectively, distinguish injury data of each part according to the above methods; 4 samples shall be removed, and then 53 samples are reserved finally.

## 4. Data Analysis

### 4.1. Rider's Injury Source Analysis of Each Part

Take the vehicle speed as the *x*-coordinate and the rider's injury value of each part as the *y*-coordinate, respectively; draw comparison diagrams concerning the rider's injury source of each part. In the diagram, the triangle and circular scatter, respectively, indicate that the injury source is from automobiles and the road pavement; the solid line and dotted line indicate trend lines of the triangle and circular scatter.

#### 4.1.1. Head

**Figure 6:**
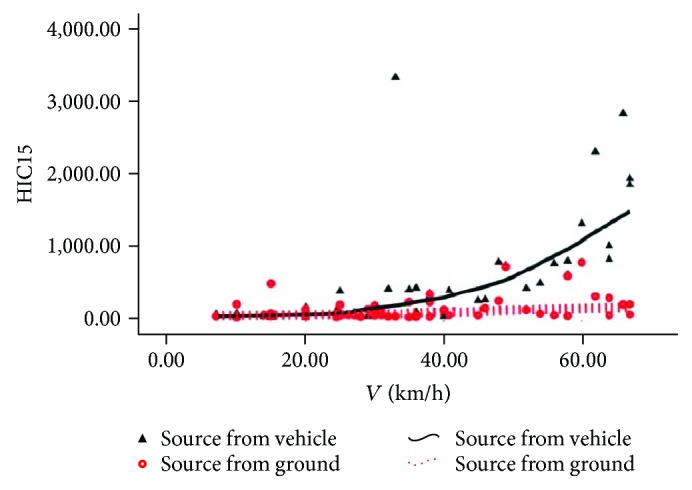
Source of head HIC15.

**Figure 7:**
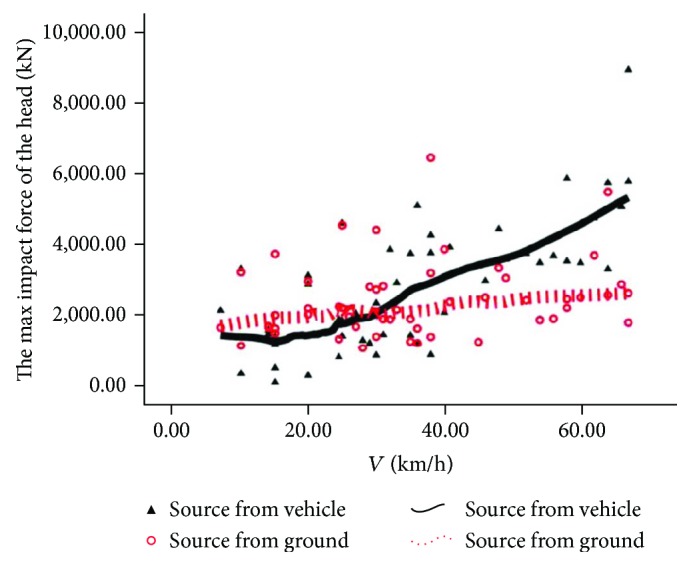
Source of head maximal striking force.

The source comparison of the rider's head HIC15 and maximum head collision force is shown in Figures 6 and 7. Upon low speed, in Figure 6, the solid line is close to the dotted line, indicating that the difference of the indirect injury value caused by automobiles and the road pavement is not obvious; in Figure 7, the dotted line is higher than the solid line, indicating that the direct injury of the rider's head is mainly from the road pavement. Upon intermediate and high speed, in Figures 6 and 7, the solid line is obviously higher than the dotted line, indicating that both indirect and direct damages of the head are mainly from automobiles. In order to make the discussion more convenient and coherent, the specific interval of the velocity will be replaced by the low speed, intermediate, and high speed. Generally, low speed is about 0 to 30 km/h, intermediate speed is about 30 to 50 km/h, and high speed is about 60 to 80 km/h

#### 4.1.2. Torso

**Figure 8:**
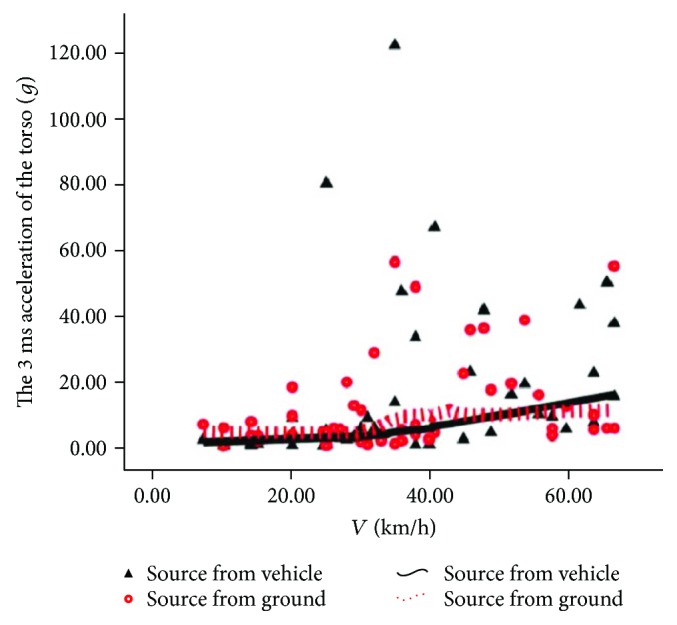
Source of injury of torso 3 ms acceleration.

**Figure 9:**
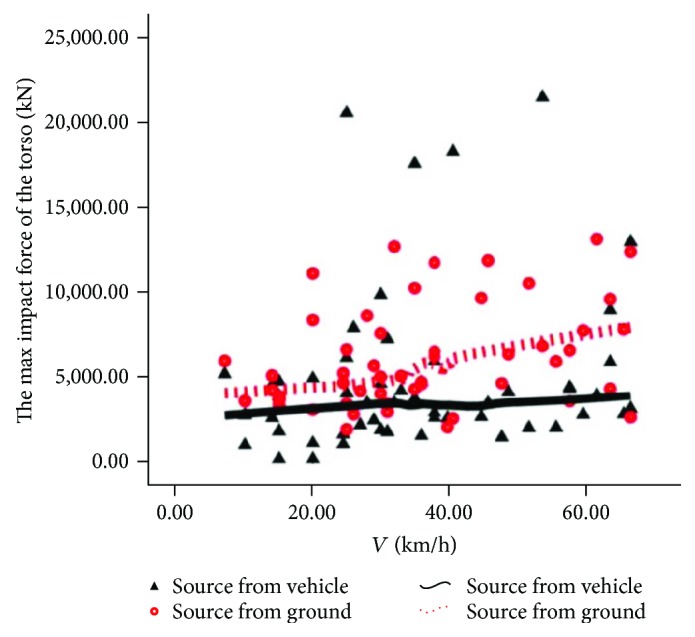
Source of torso maximal contact force.

The source comparison of the rider's torso 3 ms acceleration magnitude and maximum torso contact force is shown in Figures 8 and 9. In Figure 8, we can see that the contact ratio between the dotted line and the real line is very high upon intermediate and low speed, indicating that the main source of indirect injury to the rider's torso is not obvious at low and intermediate speed, and the main source is from automobiles at high speed; in Figure 9, we can see that the dotted line is obviously higher than the solid line, indicating that the direct injury of the rider's torso is mainly from the road pavement.

#### 4.1.3. Tibia

**Figure 10:**
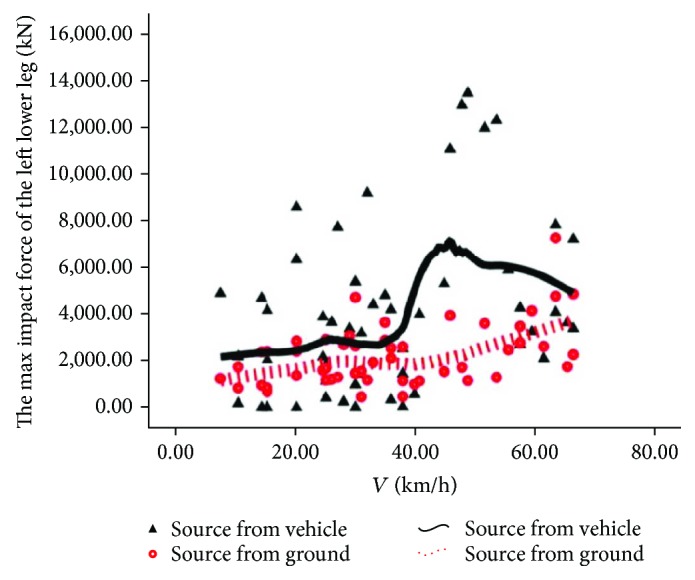
Source of injury of the lower left leg.

**Figure 11:**
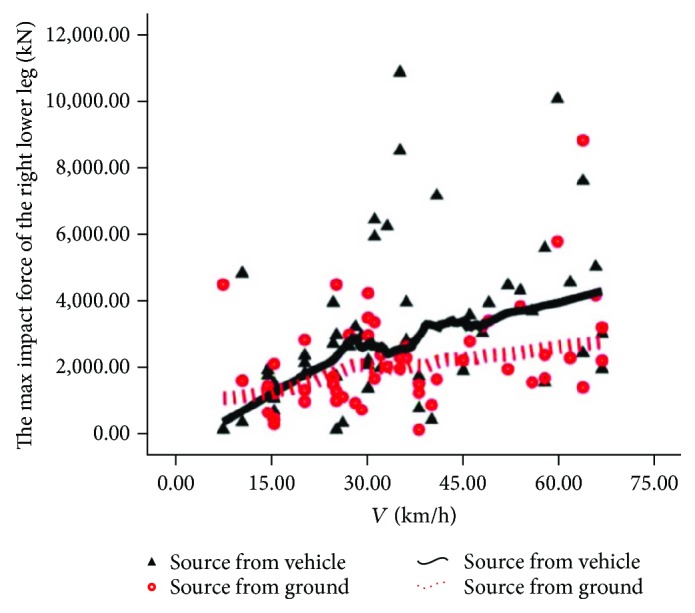
Source of injury of the lower right leg.

The source comparison of the rider's maximum tibia contact force is shown in Figures 10 and 11. In Figure 10, we can see that the solid line is above the dotted line, indicating that the injury of the rider's left leg is mainly from automobiles. In Figure 11, upon low speed, the dotted line is above the solid line, indicating that the injury of the rider's right leg is mainly from the road pavement; upon intermediate and high speed, the solid line is generally located above the dotted line, indicating that the injury of the right leg is from automobiles.

#### 4.1.4. Femur

**Figure 12:**
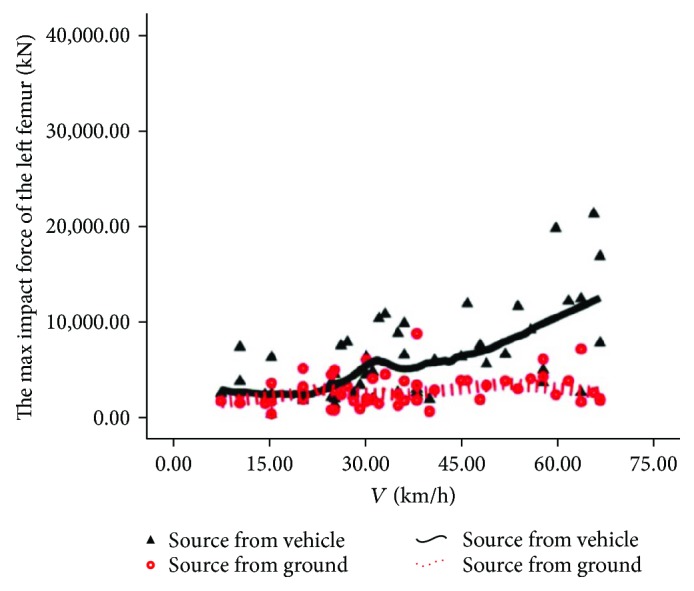
Source of injury of the left femur.

**Figure 13:**
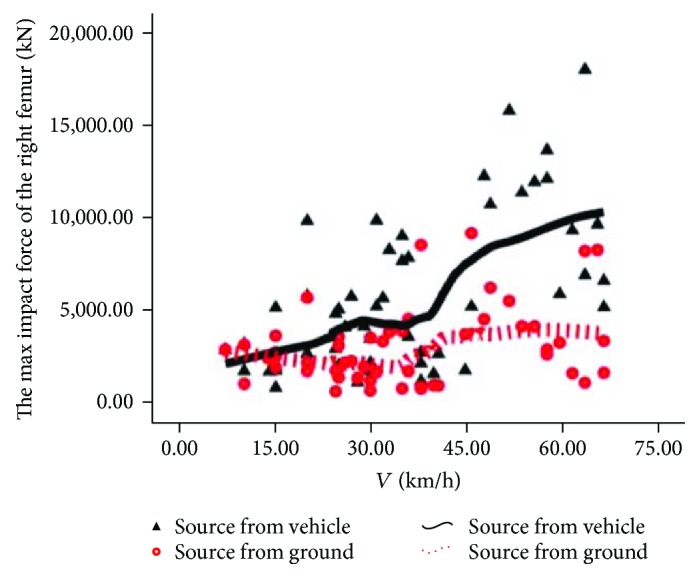
Source of injury of the right femur.

The source comparison of rider's maximum femur contact force is shown in Figures 12 and 13. In Figure 12, in addition to individual points, the solid line is generally located above the dotted line, indicating that the injury of the rider's left femur is mainly from automobiles in most cases. In Figure 13, similar to the law of the left femur, the right femur injury is from automobiles in most cases.

#### 4.1.5. Knee

**Figure 14:**
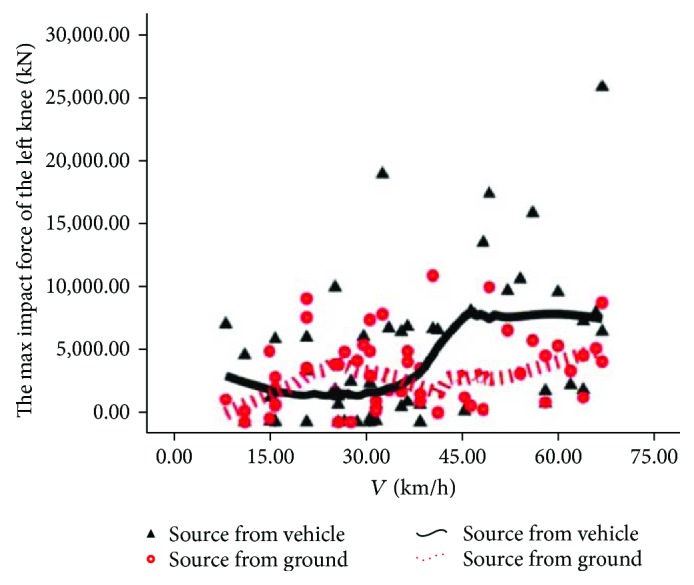
Source of injury of the left knee.

**Figure 15:**
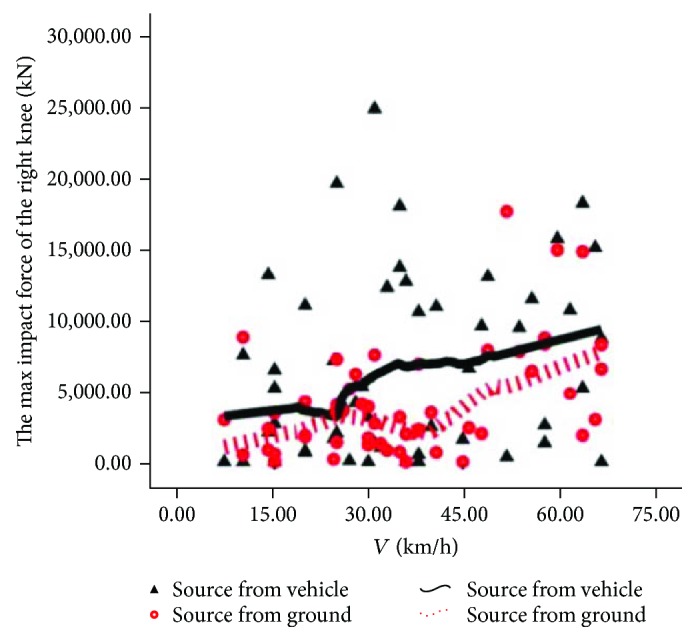
Source of injury of the right knee.

The source comparison of the rider's maximum knee contact force is shown in Figures 14 and 15. In Figure 14, we can see that 2 curves are in a staggered upward trend. Upon low speed, the solid line is above the dotted line, indicating that the injury to the left knee of the rider is from automobiles. Upon intermediate speed, the dotted line is obviously higher than the solid line, indicating that the injury to the left knee of the rider is from the road pavement; upon high speed, the solid line is above the dotted line, indicating that the injury to the left knee is from automobiles. In Figure 15, we can see that two curves are in a wavelike upward trend and the solid line is above the dotted line, indicating that the injury of the rider's right knee is mainly from automobiles.

### 4.2. Correlation Analysis of Injuries

The rider's injury correlation of each part was analyzed by the rank correlation coefficient method in the SPSS, and analysis results are shown in Table 7.

Table 7 shows stronger correlations among rider's head injury HIC15 and torso 3 ms acceleration magnitude, maximum left femur contact force, and maximum right femur contact force. There is a certain correlation between rider's torso 3 ms acceleration magnitude and maximum left femur and right femur contact forces. It shows stronger correlations between maximum collision force of the rider's left femur and maximum right femur contact force, which has a certain correlation with maximum contact force of the rider's left leg and left knee. There is a certain correlation between maximum collision force of the rider's right femur and maximum contact force of the rider's left leg and right leg. It has a certain correlation between the maximum collision force of the rider's left leg and maximum collision force of the right leg and left knee. The above has a significant statistical significance; there is a medium correlation between maximum contact force of the rider's right leg and right knee; there is no obvious correlation between maximum contact force of the rider's left knee and right knee.

## 5. Conclusion

After accident reconstruction, data acquisition, data verification, and screening of 57 car-electric bicycle accidents where riders are hit against the engine hood and thrown to the air, data obtained from the remaining 53 cases were analyzed and the following conclusions were drawn:

1. Through comparing the rider's injuries of each part in the collision process with automobiles and ground, we found that direct injuries of the head and right leg are from the road pavement upon low speed. The source laws of indirect head injuries are not obvious; upon intermediate and high speed, the injuries of the above parts are from automobiles. In most cases, injuries of the left leg, femur, and right knee are from automobiles; left knee injuries are from automobiles, the road pavement, and automobiles, respectively, upon low, intermediate, and high speed. The source laws of indirect torso injuries are not obvious upon intermediate and low speed, which are from automobiles upon high speed, while direct torso injuries are from the road pavement.
2. No high correlation was found between all parts of the injury. The largest correlation coefficient was the head-left femur and left femur-right femur, which was 0.647, followed by the head-right femur (0.638) and head-torso which was 0.617. It was found that the correlation coefficient values of the abovementioned items were very close, and it needs to be further studied whether the approach was related to the collision angle.
3. Though some interesting results were obtained, the reason why there are such phenomena was not discussed here, which deserves to be studied deeply in the future.

## Figures and Tables

**Table 1:** The comparison of rider injury in simulation and true injury.

Body	Injury criteria	Reconstructed results	Conjectured injury	True injury provided by the police
Head	HIC15 ≤ 1000 [15]	997.6	No fatal injury	Consciousness
Torso	Resultant acceleration 3 ms ≤ 60*g* [16]	22.53*g*	No fatal injury	Three fractures on the rib
Femur	<6.3 kN [17]	Left: 2303.7 N Right: 5674.5 N	No fracture of the femur	No injury of the femur
Tibia	<4 kN [17]	Left: 4781.5 N Right: 2363.2 N	Fracture of the left tibia	Open fractures on the left leg and trauma on the right leg

**Table 2:** 

Case no.	Sp (m)	Sc (m)	Sm (m)	*V* (km/h)	HIC	HIC-v	HIC-g
1	13.9	10.7	22.6	48.0	5939.0	5939.0	0.4
2	8.4	8.4	8.7	40.8	375.1	375.1	31.1
3	10.8	6.0	13.2	36.0	75.6	75.6	18.2
4	18.6	25.4	39.5	58.0	596.1	596.1	21.1
5	10.9	13.3	4.6	25.0	27.8	24.8	27.4
6	16.6	12.3	19.9	38.0	326.6	304.3	326.6
7	13.0	9.9	20.8	35.0	389.5	389.5	5.5
8	23.6	18.7	37.7	67.0	1935.0	1935.0	40.4
9	2.2	2.8	1.9	18.0	745.2	745.2	11.6
10	4.6	2.0	4.3	20.0	142.3	142.3	17.8
11	9.6	5.9	5.1	33.0	3337.0	3337.0	14.6
12	6.9	3.9	3.1	27.0	68.2	68.2	29.1
13	32.3	20.6	23.1	51.0	443.9	443.9	73.6
14	5.8	11.8	18.4	49.0	201.7	201.7	165.8
15	8.9	7.0	6.2	30.0	116.0	116.0	56.1
16	4.9	3.6	4.9	26.0	39.1	39.1	37.5
17	6.8	5.9	7.6	36.0	408.5	408.5	9.4
18	19.8	10.3	26.1	45.0	234.8	234.8	28.0
19	8.0	9.6	8.4	31.0	68.6	68.6	30.0
20	4.4	1.8	3.8	20.0	97.0	97.0	41.2
21	4.3	3.3	6.3	25.0	363.1	363.1	**32.7**
22	3.2	1.9	5.4	15.0	59.3	3.2	59.3
23	1.5	3.0	1.2	15.0	24.0	24.0	17.5
24	17.4	21.6	20.9	38.0	338.6	338.6	10.0
25	11.4	8.3	12.8	24.5	98.1	98.1	5.3
26	1.0	12.6	1.0	40.0	106.9	12.1	106.9
27	12.9	18.2	9.7	29.0	110.4	11.8	110.4
28	4.0	2.8	8.3	24.5	58.9	4.6	58.9
29	3.3	17.7	3.2	30.0	166.0	166.0	164.5
30	3.5	1.3	1.4	14.0	33.7	18.9	33.7
31	3.5	5.9	2.8	28.0	12.9	12.9	9.0
32	1.0	0.5	0.4	10.0	54.5	54.5	0.7
33	5.9	1.3	4.9	14.0	34.8	11.4	34.8
34	18.6	20.0	24.7	64.0	808.6	808.6	270.2
35	5.6	2.5	4.8	15.0	48.8	17.2	48.8
36	6.9	4.5	6.0	32.0	395.2	395.2	13.9
37	4.3	1.1	3.9	15.0	471.7	3.3	471.7
38	3.5	0.6	2.3	10.0	183.2	0.7	183.2
39	21.6	29.2	28.1	67.0	1849.0	1849.0	183.0
40	23.2	60.2	20.1	64.0	997.6	997.6	28.3
41	4.0	3.8	6.8	30.0	115.4	115.4	26.3
42	2.1	11.9	4.1	31.0	87.7	29.7	87.7
43	9.9	11.9	8.0	35.0	214.8	209.1	214.8
44	2.1	1.2	1.0	7.0	43.7	43.7	15.9
45	6.8	5.4	5.2	25.0	176.2	58.4	176.2
46	10.6	2.0	7.9	20.0	115.2	0.2	115.2
47	2.0	31.2	11.2	38.0	212.2	18.8	212.2
48	11.3	8.4	12.4	46.0	246.2	246.2	129.1
49	12.9	9.1	14.5	48.0	766.9	766.9	231.6
50	10.1	9.5	16.0	49.0	720.5	720.5	705.7
51	13.9	11.6	11.9	54.0	478.5	478.5	52.6
52	11.9	13.3	11.5	56.0	748.4	748.4	32.1
53	17.8	13.7	13.6	58.0	780.9	780.9	575.4
54	18.1	15.0	10.6	60.0	1306.0	1306.0	764.6
55	15.6	15.0	23.0	62.0	2300.0	2300.0	293.2
56	17.5	16.9	28.9	66.0	2833.0	2833.0	180.7
57	15.7	10.4	17.1	52.0	405.8	405.8	106.1

Sp: throw distance of the pedestrian; Sc: braking distance of the vehicle; Sm: throw distance of the motorcycle; *V*: velocity of the vehicle; HIC-v: HIC from the vehicle; HIC-g: HIC from the ground.

**Table 3:** 

Case no.	Head 1 (N)	Head 2 (N)	3 ms 1	3 ms 2	Torso 1 (N)	Torso 2 (N)	Femur left 1 (N)
1	2831.7	4648.0	7.2	6.3	2503.3	10502.6	1605.4
2	3927.8	2351.7	67.0	4.3	18312.0	2405.9	5727.9
3	1149.4	1581.4	2.4	1.7	1388.6	4627.2	9657.8
4	5914.5	2175.9	4.5	3.4	4178.7	3444.0	4717.8
5	1358.2	1734.9	0.6	0.3	3930.6	3285.4	1040.8
6	3761.2	6515.4	33.5	48.8	5898.5	11708.0	2268.9
7	3744.3	1197.0	13.5	0.8	3617.1	4180.9	8606.1
8	9049.5	1750.5	37.7	5.6	12957.0	2478.9	7591.1
9	6324.7	2079.9	14.4	0.5	4525.5	1463.2	20684.0
10	3109.0	1985.9	8.7	3.6	938.1	2916.4	31983.0
11	2899.0	2118.8	3.6	1.8	4082.6	4984.0	10640.0
12	2009.2	1632.3	2.6	5.3	2009.2	4059.8	7679.2
13	3743.8	2416.0	154.8	19.8	20363.0	12895.0	10944.0
14	4476.5	3515.5	7.8	267.0	3662.5	27000.0	5932.1
15	1935.6	2704.1	2.4	2.4	1736.1	4931.7	6140.1
16	1783.7	1916.6	4.7	5.6	7838.2	2654.6	7287.2
17	5128.0	1147.7	47.6	5.3	4593.3	4483.5	6296.2
18	3386.3	1185.3	2.2	22.4	2506.0	9605.6	6111.8
19	1394.8	1852.3	1.5	0.7	1609.4	2794.4	1625.4
20	2857.2	2165.8	3.8	18.2	4841.0	8313.8	1481.0
21	4615.9	2175.6	4.9	4.4	6064.6	6557.2	1659.6
22	420.1	1960.2	2.8	1.8	3741.1	3232.8	1473.7
23	1331.1	1579.2	2.0	3.2	4649.5	3522.0	3.7
24	4277.7	1332.5	5.1	3.6	2468.9	6429.2	2678.3
25	1834.5	1268.0	3.6	4.5	1483.6	4563.4	3403.4
26	2041.3	3865.8	0.6	2.2	2427.3	1889.4	1574.7
27	1147.4	2784.4	2.8	12.5	2303.8	5588.9	3083.2
28	734.4	2211.8	0.2	3.3	878.6	5140.3	1785.3
29	789.9	4425.2	5.7	1.4	9804.1	7521.2	4294.5
30	1421.3	1651.2	0.3	3.2	2437.3	5008.3	1558.2
31	1230.8	1003.0	2.2	19.6	3334.2	8565.9	2224.7
32	3305.3	1068.3	0.7	0.2	2606.6	3443.9	7133.2
33	1534.9	1611.7	0.9	7.6	4425.3	4115.2	2082.5
34	5788.4	5522.2	7.3	9.6	8908.9	9547.3	12278.2
35	1097.6	1437.4	0.8	3.2	1639.2	3715.4	2146.5
36	3861.6	1838.1	5.7	28.7	3349.4	12676.5	10194.5
37	0.0	3731.1	3.5	3.1	0.0	3768.0	6044.1
38	254.3	3209.1	0.1	5.7	828.5	3474.5	3485.2
39	5830.3	2599.7	15.4	55.3	2999.9	12353.4	16814.6
40	3297.0	2544.0	22.5	5.1	5816.4	4214.6	2303.7
41	2317.0	1339.4	2.5	11.1	4521.1	3911.8	4187.3
42	2093.9	2804.2	8.7	0.6	7174.3	3282.5	4513.3
43	1379.3	1855.5	122.8	56.4	17593.0	10208.0	2336.1
44	2098.2	1608.6	2.1	6.8	5099.1	5876.2	2190.5
45	1806.2	4558.7	80.5	2.4	20602.0	1758.2	4258.9
46	196.9	2943.5	0.3	9.4	0.0	11079.0	1737.6
47	805.2	3183.9	0.6	6.7	2779.1	6126.6	1930.0
48	2955.8	2476.1	22.8	35.7	3337.9	11835.0	11766.0
49	4453.8	3331.8	41.7	36.2	1291.2	4551.3	7345.4
50	3590.0	3041.7	4.3	17.3	4006.0	6275.5	5345.9
51	3481.7	1824.6	19.1	38.8	21538.0	6761.1	11477.0
52	3679.8	1862.3	9.9	15.9	1865.6	5848.1	8981.5
53	3529.5	2431.6	9.1	5.5	4318.7	6507.2	3356.9
54	3480.0	2476.6	5.3	12.1	2635.1	7676.1	19813.0
55	4768.7	3690.7	43.3	104.7	3836.0	13124.0	12044.0
56	5101.8	2855.3	50.2	5.6	2661.8	7755.5	21346.0
57	3747.1	2397.7	15.9	19.3	1849.2	10479.0	6368.6

Head is the contact force of the head, 1 means from the vehicle, and 2 means from the ground the same in the next. Data from cases 1, 11, 13, and 14 are removed from the manuscript.

**Table 4:** 

Case no.	Femur left 2 (N)	Femur right 1 (N)	Femur right 2 (N)	Lower leg left 1 (N)	Lower leg left 2 (N)	Lower leg right 1 (N)	Lower leg right 2 (N)
1	1220.9	9741.8	1568.6	1390.3	1624.0	2832.6	2825.9
2	2557.6	2533.0	779.2	3999.0	1146.1	7209.5	1565.7
3	3530.0	7856.4	1581.2	4212.0	2559.8	3919.1	2559.8
4	3987.3	13794.0	2776.5	2702.7	3494.9	1475.0	2315.1
5	2546.3	1414.1	1231.5	1817.9	1688.4	2915.5	1200.6
6	8562.5	2010.2	8562.5	27.8	2612.2	1669.6	1150.3
7	906.2	9063.5	3799.4	4814.4	3668.0	10963.0	2236.1
8	1431.9	6576.9	1488.3	3392.5	2274.5	1876.7	3152.3
9	2473.7	4037.0	2132.1	3284.7	1417.4	1673.0	2356.0
10	2916.4	9890.7	2060.2	6366.2	2848.1	1733.4	2775.5
11	4237.3	8286.4	3730.5	4416.4	1939.4	6239.9	1952.7
12	2990.3	5701.7	2141.7	7788.1	1302.5	2571.9	2919.4
13	2424.4	2527.7	1820.8	4453.9	2527.2	4139.6	2367.7
14	8393.1	10732.0	8032.3	2674.4	1390.7	10245.0	2756.9
15	1315.6	1590.5	3434.7	5402.6	1469.9	1280.7	2902.9
16	2060.2	4010.1	2060.2	3667.2	1218.5	210.7	1027.8
17	1441.3	3487.8	4463.2	337.0	2119.7	2761.7	2221.1
18	3591.9	1619.8	3627.8	5326.0	1542.4	1822.0	2154.4
19	1533.3	5158.0	1565.2	1434.6	459.0	5922.1	3306.8
20	1579.5	2554.9	1586.1	8651.7	2413.6	2292.9	884.3
21	442.4	1884.5	2991.3	1140.6	1175.8	1646.9	909.1
22	3304.1	1606.6	2620.6	2059.7	668.6	979.5	174.6
23	1330.9	661.5	2377.0	8.6	796.3	586.1	356.4
24	1473.0	2621.9	707.8	2528.6	477.2	1556.6	0.0
25	4227.9	4764.7	1611.9	3897.4	1587.0	3904.6	1640.9
26	287.7	1430.1	796.0	563.1	998.3	297.1	781.1
27	583.1	4056.6	1815.6	3395.8	3088.5	2891.4	623.4
28	464.6	2825.5	451.9	2182.5	1605.5	2650.2	1420.5
29	1669.4	2039.5	499.6	0.0	4726.7	2140.6	3439.5
30	1926.0	1562.1	2269.0	4694.7	953.5	1850.3	539.3
31	1377.7	955.8	1190.3	241.0	2704.0	3174.5	838.9
32	1165.9	3071.0	3039.1	173.5	1743.1	4797.7	1530.4
33	1123.1	2324.7	2443.4	0.0	2387.2	1694.3	1359.1
34	6921.0	18252.0	8218.5	7891.1	7322.4	7654.4	8895.7
35	2.9	1785.2	1777.0	0.0	2419.7	1438.8	1136.2
36	1100.0	5625.4	3226.3	9248.2	1181.5	1918.3	2317.9
37	1766.0	5094.2	3546.8	4185.4	847.0	401.3	2034.2
38	2106.4	1581.2	876.3	2185.0	834.3	224.6	1522.6
39	1608.7	5124.5	3245.5	7265.5	4875.4	2952.4	2141.6
40	1300.1	6890.5	938.7	4097.2	4781.5	2363.2	1320.5
41	5772.0	3372.6	1054.8	975.2	2651.6	1994.6	4196.3
42	3795.5	9912.8	1535.0	3197.1	1564.7	6441.1	1588.0
43	2127.1	7675.6	619.3	2804.1	2873.9	8575.8	1890.0
44	1391.2	2756.5	2770.7	4894.1	1245.7	0.0	4463.1
45	4708.6	5001.0	3481.9	413.8	2918.2	0.0	4463.1
46	4867.1	5730.5	5650.4	0.0	1371.9	2060.7	1237.1
47	3086.7	1039.1	607.7	1452.1	1148.2	678.8	1413.6
48	3574.3	5154.7	9210.5	11139.0	3951.5	3529.0	2730.9
49	1555.0	12361.0	4448.0	13036.0	1724.6	2987.1	3190.2
50	3048.9	10806.0	6196.1	13551.0	1163.2	3896.3	3356.6
51	2682.0	11466.0	4061.3	12388.0	1294.0	4274.7	3789.1
52	3767.5	12042.0	4053.8	5908.5	2483.1	3643.5	1478.9
53	5851.3	12212.0	2507.1	4278.8	2778.8	5573.9	1600.0
54	2058.6	5844.0	3152.0	3246.4	4161.4	10160.0	5774.4
55	3559.0	9369.2	1459.8	2102.7	2626.0	4523.7	2215.9
56	2290.0	9688.5	8280.3	3651.4	1758.8	5000.7	4129.1
57	3538.1	15991.0	5462.3	12058.0	3618.6	4441.9	1879.1

**Table 5:** 

Case no.	Left knee 1 (N)	Left knee 2 (N)	Right knee 1 (N)	Right knee 2 (N)
1	1606.7	1869.2	0.0	1962.3
2	7374.7	740.7	11025.0	671.2
3	7666.5	4854.6	12795.0	1962.5
4	1475.0	1563.3	1338.7	8830.4
5	1377.4	4637.2	2020.1	7315.5
6	0.0	4336.5	0.0	2300.1
7	7245.8	2490.2	13794.0	3183.4
8	26777.0	9540.3	8786.3	8337.0
9	20951.0	0.0	3439.5	10377.0
10	6792.3	8386.2	753.7	1766.3
11	7531.9	2607.1	12386.0	811.6
12	3296.2	0.0	80.0	5203.5
13	9984.6	8516.6	16633.0	2759.5
14	1438.2	4575.7	5978.9	5834.5
15	3162.6	5714.4	3164.9	1206.2
16	35.0	5652.9	3945.4	3598.6
17	1614.3	5703.7	2.6	0.0
18	864.8	1993.2	1584.9	0.0
19	97.8	909.3	1300.3	7614.1
20	4118.9	9867.4	670.5	1865.2
21	2037.0	4625.1	19714.0	1380.3
22	1545.0	1291.7	2696.7	3478.5
23	71.2	3628.5	6547.5	0.0
24	1369.2	1646.6	10653.1	2200.4
25	10765.1	2485.8	7213.0	187.2
26	7468.9	11705.3	2485.8	3500.3
27	6872.4	6211.4	5376.7	4073.7
28	2611.3	4668.2	1639.0	3396.1
29	0.0	8197.7	29802.8	1690.0
30	1949.6	5706.6	2086.9	834.5
31	0.0	4941.4	4182.5	6251.6
32	0.0	868.8	7607.9	8875.6
33	56.1	296.1	13272.2	2353.4
34	8084.1	5363.0	18314.2	14898.4
35	0.0	1420.1	5269.2	548.1
36	19854.0	8649.4	1013.8	1284.1
37	6690.3	2743.8	0.0	0.0
38	5400.1	0.0	0.0	501.6
39	7265.5	4875.4	0.0	6620.2
40	2630.2	1964.3	5273.6	1876.3
41	276.6	3752.9	0.0	3904.1
42	1412.0	1644.1	24980.0	2721.8
43	1195.2	2708.7	18109.0	706.2
44	7856.1	1822.9	0.0	2984.7
45	0.0	0.0	3506.5	4043.4
46	0.0	4374.1	11108.0	4273.7
47	0.0	2283.7	510.2	6963.2
48	8892.9	1289.6	6671.6	2406.9
49	14330.0	1000.6	9657.4	2003.1
50	18309.0	10785.0	13144.0	7941.3
51	11409.0	3925.8	9551.4	7859.4
52	16743.0	6560.2	11568.0	6479.2
53	2538.0	5341.6	2607.0	8370.4
54	10384.0	6148.4	15817.0	15012.0
55	3006.6	4141.1	10774.0	4856.0
56	8751.6	5928.8	15181.0	3004.0
57	10483.0	7370.8	347.5	17737.0

**Table 6:** The abnormal value of HIC15.

Observed value	Expectation	Standard deviation	Absolute errors	3*σ*	Results
5939	557	1001	5381	3003	Abnormal
1935			1377		Normal
3337			2779		Normal
1849			1291		Normal
2300			1742		Normal
2833			2275		Normal

**Table 7:** The rank correlation coefficient about different parts of the rider's injury.

	Head	Torso	Left femur	Right femur	Left leg	Right leg	Left knee	Right knee
Head	1.000	0.617^∗∗^	0.647^∗∗^	0.638^∗∗^	0.378^∗∗^	0.322^∗^	0.418^∗∗^	0.291
Torso	0.617^∗∗^	1.000	0.505^∗∗^	0.432^∗∗^	0.357^∗∗^	0.352^∗∗^	0.282^∗^	0.188
Left femur	0.647^∗∗^	0.505^∗∗^	1.000	0.647^∗∗^	0.524^∗∗^	0.337^∗^	0.493^∗∗^	0.209
Right femur	0.638^∗∗^	0.432^∗∗^	0.647^∗∗^	1.000	0.539^∗∗^	0.533^∗∗^	0.292^∗^	0.395
Left leg	0.378^∗∗^	0.357^∗∗^	0.524^∗∗^	0.539^∗∗^	1.000	0.401^∗∗^	0.563^∗∗^	0.111
Right leg	0.322^∗^	0.352^∗∗^	0.337^∗^	0.533^∗∗^	0.401^∗∗^	1.000	0.199	0.584
Left knee	0.418^∗∗^	0.282^∗^	0.493^∗∗^	0.292^∗^	0.563^∗∗^	0.199	1.000	0.133
Right knee	0.291^∗^	0.188	0.209	0.395^∗∗^	0.111	0.584^∗∗^	0.133	1.000

Superscript ∗∗ means significant statistical significance, superscript ∗ means general statistical significance, and no superscript means no statistical significance.
